# The Bergen COPD microbiome study (MicroCOPD): rationale, design, and initial experiences

**DOI:** 10.3402/ecrj.v1.26196

**Published:** 2014-12-29

**Authors:** Rune Grønseth, Ingvild Haaland, Harald G. Wiker, Einar Marius H. Martinsen, Elise O. Leiten, Gunnar Husebø, Øistein Svanes, Per S. Bakke, Tomas M. Eagan

**Affiliations:** 1 Department of Thoracic Medicine, Haukeland University Hospital, Bergen, Norway; 2 Department of Clinical Science, University of Bergen, Bergen, Norway

**Keywords:** COPD, control subjects, bronchoscopy, biopsies, microbiome, inflammation, remodeling

## Abstract

**Background:**

Recent methodological developments, in particular new sequencing methods for bacterial RNA/DNA, have shown that microorganisms reside in airways that do not suffer from acute infection and that respiratory microbiota might vary according to airways disease status. We aim to establish high-quality sampling methods for lower airways microbiota as well as describe the respiratory microbiome in subjects with and without chronic obstructive pulmonary disease (COPD) and to relate the microbiome to disease development, progression, and the host immune system.

**Methods:**

The Bergen COPD microbiome study (MicroCOPD) is a longitudinal study aiming to collect data from 200 subjects with COPD as well as 150 individuals without COPD. At baseline, subjects go through a bronchoscopy in which protected specimen brushes, small-volume lavage, bronchoalveolar lavage, and bronchial biopsies provide a unique chance to analyze the microbiota and the host immune system status. These variables will be related to baseline clinical parameters (lung function, smoking status, exacerbation frequency, arterial blood gases, comorbidities, and medications) as well as follow-up parameters (lung function changes, exacerbation frequency, mortality, and more).

**Results:**

Per date more than 150 bronchoscopies have been performed, equally distributed between cases and controls, with a very low complication frequency.

**Conclusions:**

MicroCOPD will provide unique data on a large material, with insight on a new field of respiratory research.

Chronic obstructive pulmonary disease (COPD) is the third most common cause of death worldwide and is expected to maintain this unattractive position at least until year 2030 (1). Tobacco smoking is the main risk factor for developing COPD, but also indoor use of biomass fuels, occupational exposures, second-hand smoking, asthma, early life infection, and tuberculosis have been shown to influence the development of this debilitating condition (2).

Although the risk factors of COPD have been fairly well characterized, we still have limited knowledge as to why only a fraction of risk factor exposed individuals develop the disease (3). Large genetic studies have shown some associations between individual genes and development of COPD (4), but there have been major issues concerning non-replication, and despite substantial efforts, identified genes only explain a marginal fraction of the COPD burden.

Previously, the healthy lung was considered sterile (5, 6). Consequently, the proposed role of microorganisms in the pathogenesis of COPD has been limited to the effect of early life acute infections and to bacterial colonization as a risk factor for recurrent acute exacerbations of COPD. However, the development of non-culture-based techniques, primarily polymerase chain reaction (PCR) and sequencing of microbial RNA (16S ribosomal RNA) (7), has shown that human beings are superorganisms with a rich microbial environment that also encompasses the lower airways (8).

The gut microbiota have been studied for more than a decade and have, for example, been associated with obesity, underweight, colorectal cancer, and inflammatory bowel diseases (9). Studies of the role of the lung microbiota in disease are at the starting line (10).

These initial studies indicate that it is possible to characterize respiratory microbiota in both COPD patients and healthy subjects and in different types of material including sputum, bronchoalveolar lavage (BAL), cytology brushes, and lung tissue (8, 11–16). Despite the low number of participants in these studies, they clearly demonstrate the complexity of the lung microbiota, with both overlap and differences in bacterial communities between healthy and diseased individuals. However, findings in different studies have been partly contradictory. For instance, the study by Erb-Downward found evidence of reduced diversity of the lung microbiota in patients with advanced COPD (8), whereas Pragman et al. found indicators of increased diversity in more severely affected individuals (14). The lack of established methods and low-powered studies might explain some of the diverging results so far.

Only two (13, 14) out of seven studies (8, 11, 12, 15, 16) published to date included more than 10 COPD patients. Besides the low number of participants, all but one of the studies collects material that is exposed to contamination from the upper airways and the oral cavity. The study by Sze et al. was based on autopsy and explanted material from transplanted patients (15), which is difficult to generalize to the larger population of COPD patients. Nevertheless, all these studies found considerable amounts of microbial DNA, unlikely to be fully explained by contamination. Furthermore, five studies include subjects without COPD (8, 13–16) and found some degree of difference in composition of the lung microbiota between individuals with and without COPD.

Although these studies imply that it is possible to characterize the lung microbiota, the need for larger studies is obvious. Whether the lung microbiota are related to actual clinical characteristics important in COPD is unknown. For instance, to answer the questions whether there is a relation between smoking habits, level of lung function, and exacerbation frequency and lung microbiota, large patient-control cohorts are needed. Furthermore, all studies so far have been cross-sectional, and no study has yet attempted to address whether the lung microbiota impact disease progression, questions for which a follow-up is necessary.

If there is a relation between lung microbiota and COPD development, the underlying mechanisms need to be addressed. To do so, it is important to also study the local and systemic host immune response. To what degree the microbial compositions are distorted or normal will require an understanding of whether the immune response locally is indicative of trying to contain or eradicate the microbials, and whether there are signs of harmful side effects of local inflammation, like remodeling of the airways or destruction of alveoli.

Finally, animal studies of allergic asthma have pointed to how the production of short chain fatty acids by gut bacteria might alter the gut and possibly lung microbiota and influence the host immune response (17). It might be necessary to include other microbiota sampling site like the oral or gut microbiota in studies of lung diseases.

The Bergen COPD microbiome study (MicroCOPD) has been designed to compare the airways microbiome in subjects with and without COPD, and study the interaction between the microbiome and the host immune response. MicroCOPD has the ambitious goal of recruiting 300 subjects with COPD and 200 subjects without COPD, gathering data with high-quality bronchoscopies with superior collection techniques to minimize contamination issues. The study is performed on well-characterized COPD patients and subjects without COPD, and participants will be followed up for at least 3 years. The present paper describes the hypothesis, objectives, and design of the study. We also report the progress made since the data collection started in April 2013.

## Hypothesis

We put forward the hypothesis that the airways contain a characteristic microbiome, which may have both beneficial and adverse effects in the airways through its interaction with the immune system. The host–microbiome interaction may be influenced by airborne exposures and other factors like epigenetic determinants, obesity, diet, and drugs. Dissimilarities in the host–microbiome interaction may explain the heterogeneity in susceptibility for COPD and the diversity in disease manifestation and progression.

## Objectives

Our main goals are to:

1. Establish a methodological standard for collection of high-quality samples of lung-diseased patients by bronchoscopy.
2. Examine the airway microbiome and the host immune response in subjects with and without COPD, and examine how it varies with smoking, age, sex, COPD disease characteristics, and comorbidities including coronary heart disease.
3. Investigate if and how the airway microbiome predicts disease progression in COPD, in terms of lung function, exacerbation rate, development of respiratory failure, cachexia, and mortality.
4. Examine the association of both genetic make-up and epigenetic effects or influences on airway microbiota, on disease progression and mortality.
5. Explore how remodeling is associated to the airways microbiome and how remodeling influences the disease progression and other outcomes in COPD.

## Study design

MicroCOPD is a 3-year prospective non-interventional study carried out at one center in Bergen, Western Norway. All participants perform a baseline visit over 1 or 2 days and are subsequently followed up for a minimum of 36 months to assess exacerbation rates and mortality. Protocol development and a pilot phase took place from spring 2011 throughout year 2012, and the first patient in the main study was included on April 11th 2013. The study is conducted in accordance with the Declaration of Helsinki and guidelines for good clinical practice. The regional committee of medical ethics approved the project (project number 2011/1307).

### Inclusion and exclusion criteria

We enroll COPD patients aged above 40 years, both ex- or current smokers, but with at least a smoking history of ≥10 pack-years. All participants are seen by a study physician prior to inclusion, and a clinical assessment is made of the diagnosis. All COPD patients have post-bronchodilation spirometry indicating airways obstruction with a forced expiratory volume in one second (FEV_1_)/forced vital capacity (FVC) ratio<0.7, according to international guidelines (18). Spirometry results will also be evaluated using the lower limit of normal (LLN)-criterion. Control subjects are ex-, never-, and current smokers, 40 years or older with a post-bronchodilator FEV_1_ of >80% of the predicted value and baseline post-bronchodilator FEV_1_/FVC≥0.7.

In some cases, the diagnostic differentiation between COPD GOLD stage 1, normal aging or even asthma may be difficult, and this is reported by the study physician on the entry form. After completion of the data collection, all difficult cases will be reviewed critically by the study team, with all data available including high-resolution computer tomography (HRCT) scans of the lungs.

Subjects are not included if they have been admitted to hospital due to COPD or taken antibiotics or oral corticosteroids the last 2 weeks, but might be included later. Subjects with symptoms of acute COPD exacerbations according to the Anthonisen criteria (19) and evaluated on site by the study physician are postponed or not included. Furthermore, we do not perform bronchoscopy on individuals that are hypoxemic and hypercapnic, have an increased bleeding risk, possess certain cardiac risk factors, or are allergic against the chosen anesthetic agents. Inclusion and exclusion criteria are presented in Table 1.

**Table 1:** Inclusion and exclusion criteria for COPD cases and controls in the Bergen COPD microbiome study (MicroCOPD)

	Common	COPD cases	Controls
Inclusion criteria			
Age	40 years or older		
Smoking habits		Ex- or current smokers	Ex-/never-/current smokers
FEV_1_/FVC		<0.70	≥0.70
FEV_1_% predicted			≥80%
Exclusion criteria			
Healthcare utilization	Antibiotic use last 2 weeks	Admission due to COPD last 2 weeks	
Respiratory symptoms	Ongoing respiratory symptom exacerbation		
Comorbidities		No restriction	Treatment for lung or airway disease
Increased bleeding risk	Double platelet inhibition, oral anticoagulant therapy, treatment with clopidogrel or ticagrelor, low molecular weight heparin treatment; total platelet count <75*10^9^, International Normalized Ratio >2.0; the presence of a coagulopathy		
Cardiac status	Cardiac valve prosthesis, known severe pulmonary hypertension, acute coronary syndrome during the preceding 6 weeks		
Arterial CO_2_ tension	>6.65 kPa		
Arterial O_2_ tension	<8.0 kPa or SpO_2_ <90% despite 3 liters/minute oxygen supply		
Other	Allergic against lidocaine or alfentanil		

COPD=chronic obstructive pulmonary disease; FEV_1_=forced expiratory volume in one second; FVC=forced vital capacity; SpO_2_=oxygen saturation as measured by a pulse oxymeter.

### Study population

COPD patients and controls are recruited from four sources:

1. The Bergen COPD Cohort Study (BCCS) which comprises 433 COPD cases and 325 controls aged 40–76 years at inclusion in 2006/07 (20), all COPD patients ever-smokers (smoked the equivalent of at least 20 cigarettes daily for 10 years) (20).
2. The GenKOLS Study, which comprised 954 COPD patients and 955 subjects without COPD in inclusion in 2002/03. This cohort is currently undergoing a follow-up at our study center, and eligible COPD patients and controls are offered simultaneous participation in the MicroCOPD study (21).
3. COPD patients from Haukeland University Hospital (HUH), Bergen, Norway. HUH is the regional hospital for about 1,000,000 subjects and has the largest pulmonary ward and outpatient clinic in Norway.
4. Volunteers that seek out the outpatient clinic after media attention.

### Data collection

**Fig. 1:**
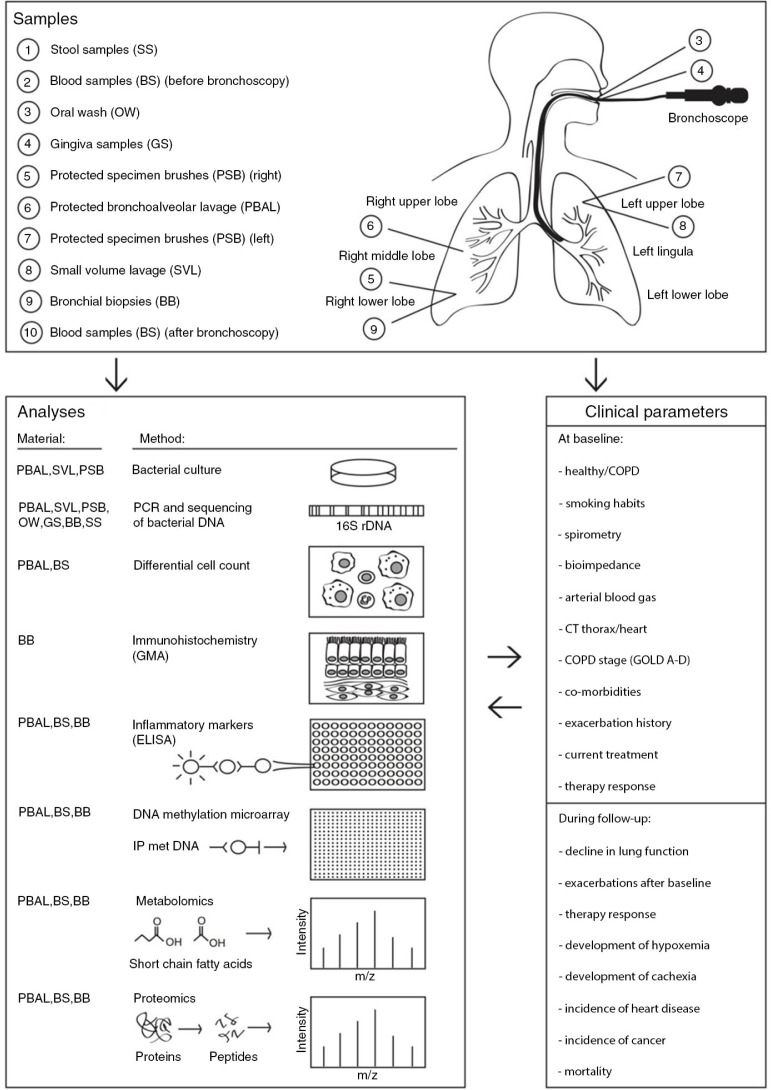
Overview of biological samples, analyses, and clinical parameters obtained from subjects included in the Bergen COPD microbiota study. Biological samples collected before, during, and after bronchoscopies, with order and location of sampling, are indicated in the figure. Stool samples (SS), blood samples (BS), oral wash (OW), and gingiva samples (GS) are collected before the bronchoscopy procedure. During the bronchoscopy, sampling is performed in the following order: three protected specimen brushes (PSB) from the right lower lobe (RLL), protected bronchoalveolar lavage (PBAL) from the right middle lobe (RML), three PSBs from the left upper lobe (LUL), small-volume lavage (SVL) from the LUL, and 4–6 bronchial biopsies (BB) from the RLL. Blood samples are also collected 1 h after the bronchoscopy procedure. The biological samples are analyzed by different methodologies; types of material used for the different analyses are indicated in the figure. Analyses include bacterial culture, PCR and sequencing of bacterial DNA (16S rDNA), differential cell count, glycol methyl acrylate (GMA) immunohistochemistry, analysis of inflammatory markers by ELISA (enzyme-linked immunosorbent assay), immunoprecipitation (IP) of methylated DNA followed by DNA methylation microarray, metabolomics, and proteomics. The results from the different analyses will be correlated to each other, in addition to clinical parameters of the patients obtained at the baseline visit and during follow-up. Examples clinical parameters are given in the figure. The bronchoscopy illustration is modified from http://www.patient.co.uk/health/bronchoscopy-leaflet.

An overview of data collected at the baseline visits is presented in Table 2. All the variables in Table 2 are collected prior to the bronchoscopies, since the performance of a bronchoscopy could impact, for instance, the spirometric measurements and HRCT scans of the lungs. The biological sampling before, during, and after the bronchoscopies is summarized in Fig. 1.

**Table 2:** Data collection at baseline in the Bergen COPD microbiome study

Variables	Collection method	Equipment	Collected by
FEV_1_, FVC	Spirometry	Viasys Vmax ENCORE	Study technicians
BMI, FMI, FFMI	Bioelectrical impedance	Bodystat 1500	Study technicians
Arterial blood gases	Radial artery puncture	Radiometer ABL 800 flex	Study technicians
Blood Pressure	Automatic blood pressure monitor	Omron HEM-757	Study technicians
%LAA, AWT	CT scan of the lungs	Siemens Somatom definition flash	Radiologists
Calcium score, stenosis grade, coronary plaque characterization	CT coronary angiography	Siemens Somatom definition flash	Radiologists
Comorbidities	Structured interview		Study physician
Smoking habits	Structured interview		Study physician
Exacerbation history last year	Structured interview		Study physician
Concurrent medications	Structured interview		Study physician

COPD=chronic obstructive pulmonary disease; FEV_1_=forced expiratory volume in one second; FVC=forced vital capacity; BMI=body mass index; FMI=fat mass index; FFMI=fat-free mass index; %LAA=% low attenuation areas; AWT=airway wall thickness; CT=computed tomography.

### Blood samples

Peripheral venous blood is drawn before the bronchoscopy, for assessment of hematology and bleeding status prior to procedure, as well as sampling of 7 ml plasma, 7 ml serum, and 7 ml of full blood. Serum is coagulated at room temperature for >30 min, followed by centrifugation at 2,500 g for 15 min at 4°C. Plasma is centrifuged immediately at 2,150 g, also for 15 min at 4°C. Both plasma and serum samples are aliquoted and stored at −80°C. One hour after the bronchoscopy both 7 ml plasma and 7 ml serum is collected again.

### Stool samples

Each participant provides two tubes with material from one stool sample – one with fresh feces and on in preservation fluid. The time between defecation and delivery at the outpatient clinic is less than 72 h. After receiving the tubes they are stored at −80°C for later DNA extraction and sequencing of the gut microbiota.

### Description of the bronchoscopic procedure

The participants are fasting at least the last 4 h before the procedure. The procedure is explained in detail to the participant, both written in the original consent form, as well as in detail by the study physician pre-procedure. All participants receive at least 400 mcg of salbutamol through a spacer before the procedure. We collect an oral wash of 10 mL phosphate-buffered saline (PBS) and gingival samples from the upper and lower interdental spaces immediately before the procedure.

An experienced study physician, assisted by a trained nurse, performs a flexible video-bronchoscopy. We favor an oral route from a posterior approach with the study subject in supine position. Topical anesthesia is provided by a 10 mg/dose lidocaine spray onto the base of the tongue and down the oropharynx and 20 mg/ml lidocaine is delivered to the vocal cords, trachea, and bronchi through a catheter. All participants are advised to accept an offer of light sedation with alfentanil 0.25–1.0 mg. Oxygen is administered to all participants through a nasal cannula during the procedure. During bronchoscopy, non-invasive blood pressure (NIBP), a three-lead electrocardiogram (ECG), and finger pulse-oxymetry are monitored.

The actual procedure is filmed at full length from the passage of the vocal cords through termination, for later quality assessment. Suction is never applied prior to passing the vocal cords – if that were to be necessary, a new bronchoscope would be found.

After the procedure, all participants are monitored for 2 h before they are allowed to eat and drink. Both immediately after the procedure, after 2 h and after 1 week, they are interviewed with a structured interview regarding side effects and discomfort.

### Bronchoscopic sampling

1. *Three protected specimen brushes (PSB) from the right lower lobe (RLL)*. The bronchoscope is extended into position, usually in the anterior basal segment (RB8), and a minimum of 10 brushing movements is performed with three different brushes. We use a double-sheathed, wax-plug protected specimen brush (Conmed, Utica, NY, USA).
2. *Fractioned BAL of the right middle lobe (RML)*. After wedging of the bronchoscope in RML, most often in the lateral segment (RB4), we instil a total of 100 ml PBS through a wax-tip protected catheter (Plastimed Combicath, prod number 58229.19) using two 50 ml syringes. Each syringe is used to aspirate immediately after installation. BAL is not performed if the measured FEV_1_<is lower than 1.0 liters and lower than 30% of predicted.
3. *Three PSB's from the left upper lobe (LUL)*. Usually sampled from the anterior segmental bronchus (LB3).
4. *Small-volume lavage (SVL) from LUL*. When the bronchoscope is in the same position as where the brushes were taken, two serial connected lavage traps are connected, and 20 ml of PBS is installed by the working channel, and thereafter suctioned into the lavage traps.
5. *Endobronchial biopsies from the RLL*. Up to six endobronchial biopsies are taken by straddling a second to fourth generation carina of the RLL carina 2–3 using a 1.8-mm disposable cupped forceps (Olympus). To minimize bleeding, 5 ml of 0.1 mg/ml adrenaline is installed before taking the biopsies.

### Analysis of the airways microbiota

PSB material: After the procedure the three brushes collected from the same site are cut off and placed in an Eppendorf tube with 1 ml PBS. After gentle vortexing of PSB, SVL, and PBAL samples they are cultured on fastidious anaerobic agars, by spread plate technique of 10 and 100 mcl to make a semiquantitative estimate of CFU upon anaerobic culture for 3 days. The cultured bacteria are frozen at −80°C on Greaves medium.

Bacterial DNA is extracted from all relevant samples (See Fig. 1) sequentially by 1) enzymatic digestion using Lysozyme, Lysostaphin and Mutanolysin followed by 2) FastDNA Spin Kit from MP Biomedicals, LLC, Solon, OH, USA. The DNA extracted by each method is pooled prior to amplification of the V3–V4 region of the 16S ribosomal RNA gene and sequencing according to the protocol for Metagenomic Sequencing Library Preparation for the Illumina MiSeq System (Part # 15044223 Rev. B). Sequencing is performed by indexing 96 samples in each setup. Identification of microbes is done using ‘Green genes’ or databases updated with respect to type strains and classification of uncultured microbes.

### Inflammation and host response

For analysis of cellular composition and inflammatory markers, the BAL fluid is immediately placed on ice and processed within 1 h after sampling. The fluid is filtered through 100 µm nylon cell strainers before determination of total cell count (Burker counting chamber) and preparation of cytospins for differential cell count (May Grunwald Giemsa staining). The remaining BAL fluid is centrifuged, and the supernatant is alliquoted and stored at −80°C for future analysis of inflammatory markers.

Plasma and BAL will be examined for inflammatory markers by ELISA (enzyme-linked immunosorbent assay), current candidates include cytokines TNFα, IL-6, IL-8, IL-17, IL-18, IL-22, IP-10, MIG) and the antimicrobial peptides NGAL, SLPI, LL-37. In addition, large-scale discovery proteomics experiments will be performed on BAL samples in order to identify and quantify biomarkers associated with distinct microbiome signatures. Selected markers identified in the proteomics studies will be validated by targeted quantitative proteomics approaches in a large number of single BAL samples, as well as in serum samples for future clinical use.

Up to three bronchial biopsies are embedded in glycol methyl acrylate (GMA) for investigation of influx of inflammatory cells (mast cells, neutrophils, macrophages, CD3+, CD4+, CD8+, eosinophils), inflammatory markers and remodeling of the airways (immunohistochemistry). The remaining bronchial biopsies are snap frozen in liquid nitrogen for later analysis of inflammatory markers, proteomics, epigenetics and metabolomics. Epigenetics and metabolomics analysis will be performed also for BAL and blood samples. The main focus for epigenetics will be global DNA methylation (microarray) and gene-specific DNA methylation (candidate genes IREB2, FAM13A, HHIP), while metabolomics studies will focus on analysis of short chain fatty acids (acetate, propionate, butyrate).

## Preliminary results

We performed 160 bronchoscopies of 77 COPD patients and 78 control subjects between the 11th of April 2013 and 30th of June 2014. Five participants were asthmatics, and eligible for a later add-on study on asthma. There was a low frequency of serious complications resulting in four hospital admissions, of which three were likely related to the procedure (one pneumonia, one non-infectious febrile reaction and one case of bronchospasm in an asthma patient. The fourth admission was due to a non-confirmed transient ischemic attack. No long-term negative effect of these incidents is known.

Among both COPD patients and controls there were more included men than women. Control subjects were younger than cases (66 vs. 69 years, *p*=0.11). The mean FEV_1_ in% of predicted among the cases was 53% (lowest 17%). For 13 cases and seven controls we have incomplete microbial procedures – that is not sampling both brushes and lavage from both lungs. In 11 cases, the subjects were judged unfit for BAL by the study physician due to low FEV_1_. During one procedure the suction valve malfunctioned, and during one procedure bleeding obscured the vision for the bronchoscopist in the start/end of the procedure. For the remaining seven subjects, the bronchoscopy was terminated early due to excessive cough or indication of subject discomfort. Due to technical issues with the fixation of the biopsies, we started to collect endobronchial biopsies in May 2014.

## Discussion

Experiences from the first 14 months of data sampling in the MicroCOPD study have demonstrated the feasibility of a large-scale single-site study including also severely affected COPD patients (FEV1<30%) as well as control subjects. There have been few serious complications. Extensive sampling of the airways is attainable and desirable when aiming to investigate associations between the airways microbiome and the host immune system.

Compared to previous studies, MicroCOPD will have an unprecedented large number of participants, increasing the external validity of the results and minimizing the risk of committing a type II error. Our one-site design minimizes sampling differences and removes geographical origin as a confounder. By presenting a comprehensive description of our recruitment and sampling procedures, we hope to facilitate comparison with future studies.

When designing the study we paid particular attention to the issue of possible contamination. In the seven studies including both COPD patients and subjects without COPD (8, 11–16), there is large variation in how samples were collected. Four of the studies have collected bronchoscopic samples by BAL (8, 12, 14, 16) and one by conventional cytology brushes (11). None of these studies reported use of protected catheters and presumably have performed the procedure through the bronchoscope's working channel, although it is known that this probably carries a significant bacterial load from the oral cavity, oropharynx, and trachea (22). Molyneaux et al. collected induced sputum (13), which is also exposed to contamination from the superior parts of the airway and oral cavity, without providing any specificity regarding sampling site in the lung. The study by Sze et al. gathered autopsy and explant tissue (15), where the impact of extracting tissue on contamination is not known.

The issue of contamination will be present in all studies gathering samples of the lung microbiota from live subjects. There is always the possibility of the bronchoscope carrying microorganisms from more superior parts of the bronchoscopic route to the sampled sites, although this chance is likely to have been considerably reduced by not using suction before passing the vocal cords, and most importantly by utilizing protected catheters for BAL and bronchial brushings. By sampling from both the oral and gingival microbiota, we also have the possibility to apply methods to adjust for carry-over. Other authors have emphasized the large variation of lung microbiota *within* the lungs (8). As the ventilation to circulation ratio varies greatly between the basal and apical areas of the lungs, it is likely that different microbials will have different predilections. We have chosen to sample the RLL and the LUL, emphasizing inter-individual comparison.

Although next generation sequencing methods clearly represent scientific progress, the link between finding microbial rRNA-residues and clinically relevant live microorganisms is less straightforward. In our design we have applied traditional culture-based techniques as well as 16S-rRNA sequencing methods, in order to shed light on the relation between new research tools and established methods in clinical respiratory medicine.

Participants also give detailed information regarding their medical history as well as supplementary tests like spirometry, body impedance, HRCT including a CT angiography of the coronary vessels. Combined with the large number of participants, this should enable MicroCOPD to relate the airway microbiota to major COPD phenotypes. Determination of cellular composition and inflammatory markers in BAL samples will provide information on microbiome–host interactions, while the application of large-scale proteomics and metabolomics studies on the bronchoscopic samples adds a broad and translational perspective to our research.

Finally, we intend for MicroCOPD to be a longitudinal study, providing a unique association with prospective outcomes as acute exacerbations of COPD and mortality. A large fraction of our participants have been taking part in previous studies as well, implying that for sub-samples there will be available comprehensive, unique information on, e.g. occupational history, living conditions, and various lung function measurements.

All research is a compromise between an ideal design and available resources. Our protocol includes COPD subjects that are, or have been, tobacco smokers. More strength would have been added to the study if we had a group of never-smokers with COPD. However, never-smoking COPD subjects are rather rare and thus more difficult to recruit and the costs would have exceeded our given limits. Furthermore, inclusion of never-smokers would have made smoking habit the main predictor variable when comparing groups. Residual confounding from failing to fully adjust for this difference might over-shadow novel findings.

In an introductory lecture to the Thomas L. Petty Aspen Lung Conference, ‘The Lung Microbiome: A New Frontier in Pulmonary Medicine’, James Beck pointed out five main objectives for future lung microbiota studies – standardized methods, large- well-characterized studies with both cases and controls, repeated measurements, long-term follow-up and studies of the gut–lung-axis in pulmonary diseases (23). We hope to advance the field of knowledge of all these topics.

To conclude, MicroCOPD aims to be the world's largest single-site study of the airways microbiome. Designed with comprehensive, high-quality sampling by bronchoscopy of COPD patients and controls without CODP, combined with follow-up of hard end-point outcomes will likely provide insights of immense value as to the importance of the airways microbiome.
